# NEMO Inhibits Programmed Necrosis in an NFκB-Independent Manner by Restraining RIP1

**DOI:** 10.1371/journal.pone.0041238

**Published:** 2012-07-26

**Authors:** Marie Anne O’Donnell, Hidenori Hase, Diana Legarda, Adrian T. Ting

**Affiliations:** Immunology Institute, Mount Sinai School of Medicine, New York, New York, United States of America; Roswell Park Cancer Institute, United States of America

## Abstract

TNF can trigger two opposing responses: cell survival and cell death. TNFR1 activates caspases that orchestrate apoptosis but some cell types switch to a necrotic death when treated with caspase inhibitors. Several genes that are required to orchestrate cell death by programmed necrosis have been identified, such as the kinase RIP1, but very little is known about the inhibitory signals that keep this necrotic cell death pathway in check. We demonstrate that T cells lacking the regulatory subunit of IKK, NFκB essential modifier (NEMO), are hypersensitive to programmed necrosis when stimulated with TNF in the presence of caspase inhibitors. Surprisingly, this pro-survival activity of NEMO is independent of NFκB-mediated gene transcription. Instead, NEMO inhibits necrosis by binding to ubiquitinated RIP1 to restrain RIP1 from engaging the necrotic death pathway. In the absence of NEMO, or if ubiquitination of RIP1 is blocked, necrosis ensues when caspases are blocked. These results indicate that recruitment of NEMO to ubiquitinated RIP1 is a key step in the TNFR1 signaling pathway that determines whether RIP1 triggers a necrotic death response.

## Introduction

The NFκB Essential Modifier (NEMO) is a signaling adaptor that is critical for the activation of the NFκB pathway by a multitude of receptors [1]. Receptor ligation leads to recruitment of NEMO to intracellular complexes and NEMO in turn forms a scaffold for the kinases IKKα and IKKβ. Once activated, the IKK complex phosphorylates the inhibitor of NFκB protein (IκBα), which leads to the ubiquitination and degradation of IκBα via the proteasome. Degradation of IκBα releases NFκB transcription factors that translocate to the nucleus and direct gene expression. The specific signaling molecules and events required for activation of the IKK kinase complex by NEMO vary between different receptors. In the case of Tumor Necrosis Factor (TNF), one of the early steps required for activation of NFκB is the recruitment of the adaptor protein RIP1 to the cytoplasmic death domain of TNFR1 [2], [3], [4], [5]. RIP1 bound to TNFR1 is modified with non-degradative ubiquitin chains [6] by the E3 ligases TRAF2, cIAP1 and cIAP2 [7], [8], [9]. NEMO contains two ubiquitin binding domains that specifically recognize these non-degradative ubiquitin chains [10]. NEMO binds to ubiquitinated RIP1 in the TNFR1 complex in a stimulus-dependent manner and this is a crucial step in the activation of the IKK complex by TNF [11], [12]. Activation of NFκB by RIP1 and NEMO can enhance cell survival because NFκB drives expression of pro-survival genes such as cFLIP, Bcl2 family members and the E3 ligases TRAF2, cIAP1 and cIAP2 [13], [14]. However, TNF can also trigger cell death responses: the signaling events that determine whether TNFR1 ligation results in cell survival or cell death are just beginning to be untangled. We have recently shown that in T cells, activation of NFκB is a relatively late pro-survival checkpoint in the TNFR1 pathway [15]. In addition to its role in the later NFκB-mediated survival program, NEMO also has an early pro-survival function that does not require activation of NFκB [16]. Prior to the NFκB-dependent pro-survival activity of NEMO coming into effect, the binding of NEMO to ubiquitinated RIP1 prevents RIP1 from binding Caspase 8 and initiating cell death by apoptosis. This early pro-survival activity of NEMO whereby it restrains the death-inducing activity of RIP1 is a post-translational regulatory mechanism that is not dependent on transcription of pro-survival genes. While earlier studies have largely described RIP1 as a survival-signaling molecule in the TNF response, more recent studies have shown RIP1 to also be a death-signaling molecule. This death-signaling role for RIP1 is only revealed when its ubiquitination is disrupted [7], [8], [15] or when its binding partner NEMO is absent [16]. Therefore, NEMO was revealed to have a surprising new function in ensuring cell survival that extends beyond its original nomenclature. Based on these studies, we have recently proposed that in TNFR1 signaling, there are two cell death checkpoints [17]. The first checkpoint consists of RIP1 ubiquitination and binding to NEMO to prevent RIP1 from interacting with CASPASE-8. The RIP1-NEMO association then leads to IKK activation and induction of the second checkpoint whereby NFκB up-regulates the expression of survival genes. The first checkpoint provides a transient protection from cell death, whereas the second checkpoint subsequently provides a long-lasting genetically programmed protection from death. If either checkpoint fails, then the cells succumb to TNF-induced cell death.

To date, disruption of the first cell death checkpoint following TNF stimulation has been shown to lead to caspase-dependent apoptosis, the most well characterized cell death pathway. However, recent reports indicate that TNF can remain true to its original name. If caspase activity is blocked, the kinase RIP1 triggers a novel program of cell death with necrotic morphology [18], [19], [20], [21]. Whether disruption of the first cell death checkpoint also regulates entry into the necrotic pathway remains unclear. In this study, we report that NEMO-deficient T cells, which have a disruption in the first checkpoint, are more sensitive to RIP1-dependent programmed necrosis when compared to NEMO-sufficient cells. The ability of NEMO to prevent RIP1-driven programmed necrosis does not require transcription of NFκB-dependent genes; instead it is mediated by the ability of NEMO to bind ubiquitinated RIP1. Therefore, NEMO has very potent pro-survival activity: in addition to restraining RIP1 from driving apoptosis, NEMO also prevents RIP1 from instigating programmed necrosis.

## Results

### NEMO-deficient Cells are More Sensitive to TNF-induced Programmed Necrosis than NFκB-deficient Cells

**Figure 1:**
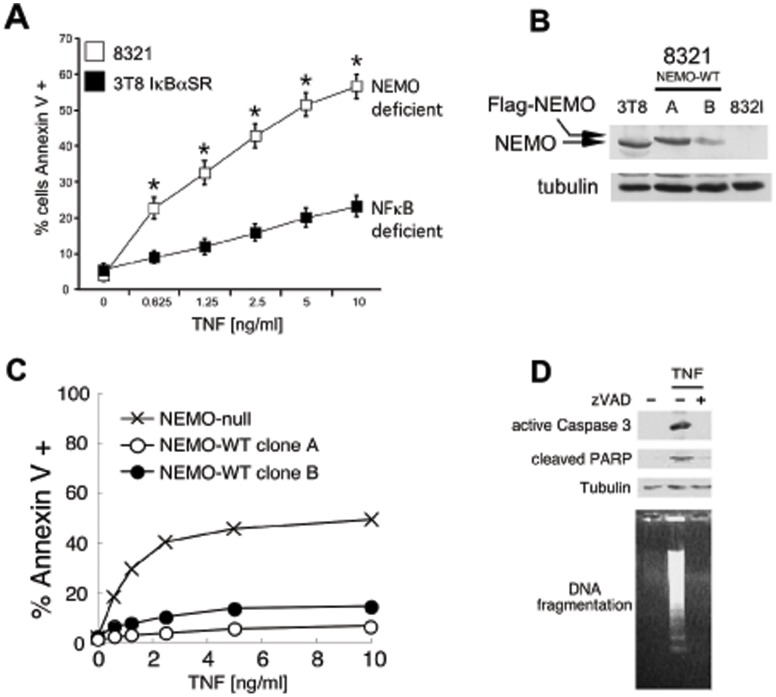
NEMO does not require NFκB to inhibit caspase-independent cell death. (A) NEMO-deficient (8321) cells and NFκB-deficient (3T8 IκBαSR) cells were co-incubated with 10 µM zVAD-fmk pan-caspase inhibitor and different doses of TNF for 24 h. Cell death was quantified by Annexin V staining and flow cytometry. The mean and SEM of six independent experiments is shown, * denotes *p*<0.05. (B) Immunoblot indicates the amount of NEMO protein expressed by the parental 3T8 cell line and the two reconstituted clones. (C) Reconstitution of NEMO-deficient cells with NEMO prevents caspase-independent cell death as shown by a TNF dose response of cell death (performed as described in (A) in two clones of NEMO-expressing cells compared to NEMO-deficient cells. (D) Immunoblot of SDS-soluble lysates from NEMO-deficient cells co-incubated with 10 µM zVAD and 10 ng/ml TNF for 24 h indicates that Caspase 3 and PARP cleavage does not occur in NEMO-deficient cells dying in the presence of pan-caspase inhibitor. Genomic DNA was electrophoresed and visualized with ethidium bromide. All cell lines shown in Figure 1 are unable to activate NFκB either due to NEMO deficiency or expression of the IκBαSR.

We have previously described an NFκB-independent function for NEMO in blocking TNF-mediated apoptosis [16]. NEMO-deficient Jurkat T cells (clone 8321) activate more caspases and die more rapidly than wild-type parental cells (clone 3T8) rendered sensitive to apoptosis by expression of the IκBα super-repressor (IκBαSR), a potent inhibitor of NFκB-mediated transcription [22]. In addition to classical, caspase-driven apoptosis, TNF can also trigger caspase-independent programmed necrosis. In this study we compared the cell death sensitivity of NEMO-deficient T cells to that of the wild-type parental T cell clone transfected with the IκBαSR (3T8/IκBαSR) when stimulated with TNF in the presence of the pan-caspase inhibitor zVAD-fmk. 3T8/IκBαSR cells undergo a low but discernable level of cell death in response to TNF when caspase activity is inhibited (Figure 1A), consistent with previous published observations that NFκB-blockade sensitizes cells to caspase-independent cell death [23], [24]. Strikingly, NEMO-deficient 8321 cells display enhanced sensitivity to cell death in the presence of caspase inhibitors when compared to 3T8/IκBαSR cells after 20 h of stimulation with TNF (Figure 1A). As a control, the sensitivity of both cell lines to cross-linking of the FAS receptor was assessed. Cell death in 8321 and 3T8/IκBαSR cells is equivalent when the FAS death receptor is triggered, and FAS-mediated cell death is completely blocked by zVAD-fmk (Figure S1) in both cell lines. Thus the NEMO-deficient cell line has enhanced sensitivity to caspase-independent cell death specific to the TNF response. Both the 8321 and the 3T8/IκBαSR cells are completely devoid of NFκB-mediated gene transcription [16], therefore, the greater sensitivity of the NEMO-deficient 8321 cell line to TNF-mediated cell death in the presence of caspase inhibitor suggests that NEMO provides an additional pro-survival signal to prevent non-apoptotic cell death independent of the NFκB transcriptional program.

To test this hypothesis, the 8321 cell line was transduced with either bacterial glutathione-s-transferase as a control protein (NEMO-null) or wild-type NEMO (NEMO-WT). 8321 cells encode a heterologous NFκB-driven Thy1 reporter gene. Two clones of cells that express NEMO-WT were established that display Thy1 expression after TNF treatment as a result of NFκB activation (Figure S2A), confirming reconstitution with functional NEMO. All three cell lines were further transfected with the IκBαSR to probe the NFκB-independent pro-survival function of NEMO. The three cell lines (NEMO-null/IκBαSR and NEMO-WT/IκBαSR clone A and B) were treated with increasing concentrations of TNF for 20 h in the presence of zVAD-fmk and cell death was examined by Annexin V staining. NEMO-null/IκBαSR cells displayed much greater sensitivity to TNF-induced caspase-independent cell death than the two clones of NEMO-WT/IκBαSR (Figure 1B), confirming that expression of NEMO (Figure 1C) prevents caspase-independent cell death independently of NFκB. To further support these results, a time-course experiment was conducted to examine the kinetics of caspase-independent cell death in NEMO-deficient T cells (Figure S2B). Enhanced cell death that occurs in NEMO-deficient T cells after 4 h of TNF treatment is predominantly caspase-dependent apoptosis and this is blocked by zVAD-fmk. The ability of zVAD-fmk to prevent TNF-induced cell death in the NEMO-deficient T cells diminishes between 8 h and 24 h, suggesting that caspase-independent cell death proceeds more slowly than apoptosis.

Jurkat T cells have been used extensively to study classical caspase-dependent apoptosis, as well as non-classical caspase-independent cell death following death receptor ligation. The latter death process has been termed programmed necrosis or necroptosis and it is characterized by the lack of hallmarks of classical apoptosis such as PARP cleavage and DNA laddering. The NEMO-deficient cells are likely dying from programmed necrosis in the presence of zVAD-fmk and to confirm this, we next examined the effect of caspase blockade on several classical features of apoptosis. NEMO-deficient cells display extensive DNA laddering when treated with TNF alone and this is abrogated by zVAD-fmk (Figure 1D, lane 2 versus lane 3). Similarly, processing of the executioner Caspase 3 and its substrate PARP are readily detectable in the lysates from NEMO-deficient cells stimulated with TNF alone but is abrogated by zVAD-fmk (Figure 1D). Despite the lack of PARP cleavage or DNA laddering, these cells are still dying as assayed by Annexin V staining (Figure 1A, 1C and S2B) or by additional assays of cell viability including the MTT, LDH release and propidium iodide exclusion assays (Figure S3). Although traditionally considered to be an event specific to the early stages of apoptosis and not necrosis, exposure of phosphatidyl serine on the outer face of the plasma membrane occurs during both pharmacological and physiological induction of non-apoptotic cell death [25], [26], [27]. Therefore, caspase blockade during TNF stimulation of NEMO-deficient T cells triggers programmed necrosis that does not display any of the usual hallmarks of apoptosis.

**Figure 2:**
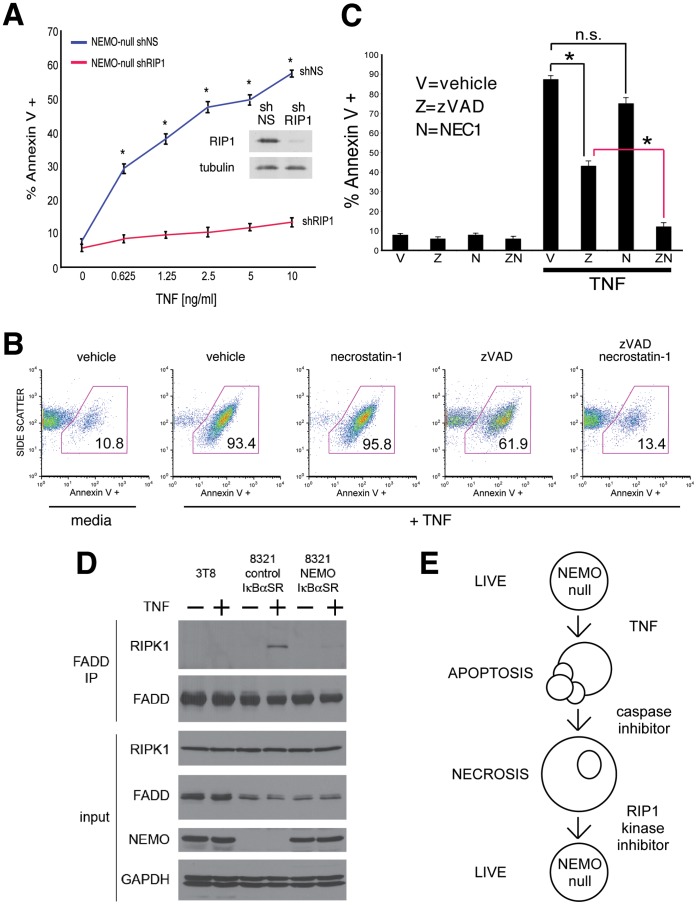
NEMO-deficient cells undergo RIP1 kinase-dependent programmed necrosis. (A) 8321 NEMO-null cells transduced with retrovirus encoding non-targeting (shNS) or RIP1-targeting short hairpins (shRIP1) were co-incubated with 10 µM zVAD caspase inhibitor and different doses of TNF for 24 h. Cell death was quantified by Annexin V staining and flow cytometry. The mean and SEM of three independent experiments is shown, * denotes *p*<0.05. Immunoblot indicates efficient knockdown of RIP1 protein in shRIP1 cells. Data shown is representative of similar results obtained with two different RIP1-targeting hairpins. (B) 8321 NEMO-null cells were pre-treated for one hour with 10 µM zVAD and/or 30 µM Necrostatin-1 as indicated, and then stimulated with 10 ng/ml TNF for 24 hours. Cell death was quantified as described in (A). The number inside the polygon gate indicates the percentage of cells that stain with Annexin V. (C) The bar chart displays the mean percentage and SEM from three independent experiments of Annexin V staining cells after 24 h of culture in vehicle, zVAD, Necrostatin-1 or a combination of zVAD and Necrostatin-1 either in the presence or absence of 10 ng/ml TNF for 24 h, * denotes *p*<0.05. (D) The parental NEMO-sufficient cell line 3T8 and NEMO-deficient 8321 cells transduced with the IκBαSR and reconstituted with either a control protein or NEMO-WT were treated with 10 µM zVAD and then stimulated with 10 ng/ml TNF for 10 hours. The necrosome was isolated by immunoprecipitation with anti-FADD and then immunoblottted for RIP1. A sample of the lysate was blotted for RIP1, NEMO, FADD and GAPDH as a loading control. (E) These data demonstrate that NEMO-null cells preferentially undergo apoptosis and caspase blockade is required for entry into RIP1 kinase-dependent programmed necrosis, which is summarized in the cartoon.

The kinase activity of the signaling adaptor RIP1 is a critical requirement for cell death by programmed necrosis [20], [21], [24]. Our previous study indicated that NEMO prevents RIP1 from functioning as a pro-death signaling molecule in the apoptosis pathway [16]. Based on these prior studies, we hypothesized that the death occurring in NEMO-deficient T cells when zVAD-fmk is present is likely to be RIP1-dependent programmed necrosis. To test this hypothesis, we stably transfected NEMO-deficient 8321 cells with a retroviral vector encoding a hairpin targeting RIP1 (8321/shRIP1) or a non-targeting control (8321/shNS). Knockdown of the endogenous RIP1 protein was confirmed by western blot and the sensitivity of the two cell lines to TNF-mediated cell death in the presence of caspase blockade was examined by Annexin V staining. RIP1 knockdown prevented NEMO-deficient T cells from undergoing caspase-independent cell death whereas the non-targeting hairpin had no effect, confirming the hypothesis that RIP1 triggers programmed necrosis in NEMO-deficient cells (Figure 2A). To further confirm these results, NEMO-deficient cells were stimulated with TNF and the kinase activity of RIP1 was inhibited with Necrostatin-1, a very specific inhibitor of RIP1’s kinase activity [28], which was initially identified for its ability to prevent TNF-triggered necrosis [29]. Necrostatin-1 alone had no inhibitory effect on the TNF-triggered cell death response in NEMO-deficient cells, which suggests that the death response in this situation is not programmed necrosis (Figure 2B, panel 2 versus panel 3). In contrast, zVAD-fmk only partially inhibited the death response, which was completely inhibited upon the addition of Necrostatin-1 (Figure 2B, panel 4 versus panel 5). We confirmed that Necrostatin-1 has no significant effect of TNF-induced cell death in the absence of zVAD-fmk, but blocks all cell death in the presence of zVAD-fmk (Figure 2C). During necrosis signaling, RIP1 is required for the activation of the related kinase RIP3 [30], [31], [32]. Knockdown of RIP3 partially limited TNF-induced programmed necrotic cell death in zVAD-treated NEMO-null cells, further confirming that loss of NEMO sensitizes cells to programmed necrosis (Figure S4). We next examined the effect of loss of NEMO protein on recruitment of RIP1 to the FADD necrosome. The necrosome from NEMO-null cells contain significantly more RIP1 than the necrosome from NEMO-null cells expressing NEMO-WT and the IκBαSR following TNF stimulation in the presence of zVAD-fmk (Figure 2D). Therefore, the presence of the NEMO protein, but not NFκB-driven gene transcription, can limit the amount of RIP1 protein recruited to the FADD necrosome when apoptosis is blocked. This suggests that the apoptotic response in the NEMO-deficient cells, which we have previously shown to be mediated by a RIP1-CASPASE-8 interaction [16], switches to a RIP1-dependent necrotic response when caspases are blocked, as outlined in the cartoon (Figure 2E). Thus in addition to inhibiting apoptosis, NEMO also inhibits the kinase RIP1 from triggering programmed necrosis and this pro-survival activity of NEMO does not require activation of NFκB.

### Ubiquitination of RIP1 is Required for NEMO to Prevent Programmed Necrosis

NEMO binds RIP1 in a ubiquitin-dependent fashion upon TNFR1 ligation. Non-degradative ubiquitination of RIP1 on lysine 377 [11], [12] by the E3 ligases TRAF2 [9], cIAP1 and cIAP2 [7] occurs rapidly upon TNF treatment and these ubiquitin chains form a docking site for a ubiquitin-binding motif on NEMO [11], [33]. We have previously reported that the binding of NEMO to ubiquitinated RIP1 prevents RIP1 from binding Caspase 8 and instigating cell death by apoptosis [16]. Based on the observation that the interaction of NEMO with ubiquitinated RIP1 prevents RIP1 from acting as a pro-death molecule in the apoptotic pathway, we reasoned that a similar mechanism would underlie the ability of NEMO to prevent RIP1 from initiating programmed necrosis.

**Figure 3:**
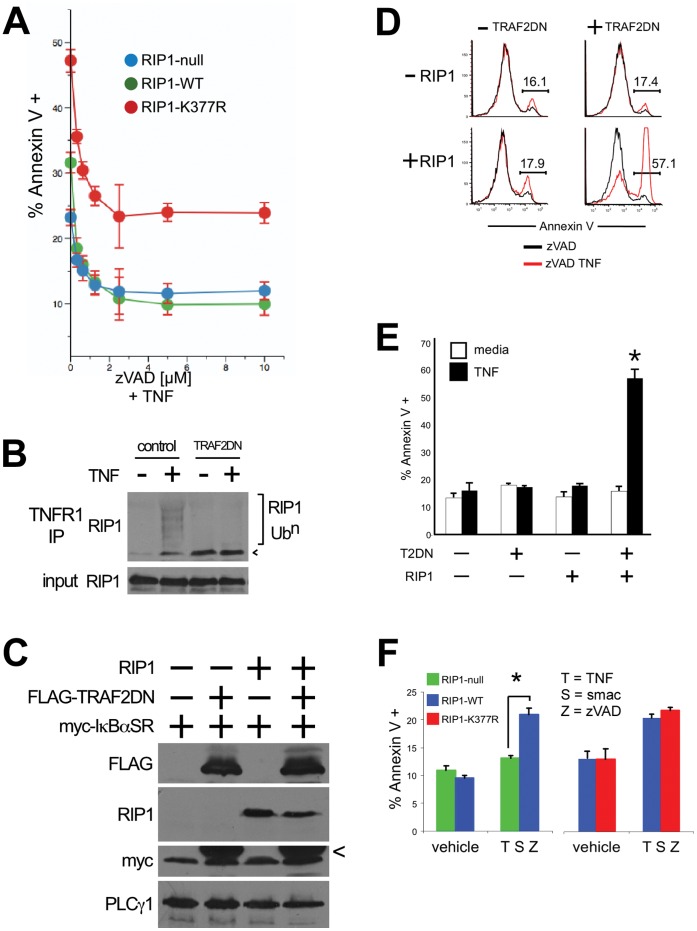
Ubiquitination of RIP1 prevents programmed necrosis. (A) RIP1-null Jurkat T cells were transduced with either a control protein, RIP1-WT or RIP1-K377R. Cells were pre-treated for one hour with different doses of zVAD and then stimulated with 10 ng/ml TNF for 24 hours. Cell death was quantified by Annexin V staining and flow cytometry. (B) Parental 3T8 Jurkat T cells were transduced with either a control protein or the TRAF2 dominant negative (TRAF2DN). Cells were stimulated with 100 ng/ml TNF for 5 minutes and the TNFR1 complex was immunoprecipitated and subject to western blot with antibodies specific to RIP1. A sample of the lysate before immunoprecipitation (input) was blotted for RIP1 as a loading control. (C) RIP1-null cells were transduced with a control protein (- RIP1) or RIP1-WT (+ RIP1) and then subsequently transduced with a control protein (- TRAF2DN) or the TRAF2DN protein (+ TRAF2DN). All four cell lines were transduced with the IκBαSR. Expression of all transgenes was confirmed by western blot (< marks remaining signal from the TRAF2DN blot) (D) The cells in (C) were pre-treated with 10 µM zVAD for one hour and stimulated with 10 ng/ml TNF. Cell death was measured after 24 hours as described in (A). Representative histograms are shown in (D): the number above the gate indicates the percentage cells that stain with Annexin V after TNF treatment. (E) The bar chart displays the mean percentage of Annexin V staining cells and the SEM of three independent experiments performed as described in (D), * denotes *p*<0.05. (F) Bar chart shows programmed necrosis in RIP1-null cells reconstituted with either a control protein, RIP1-WT or RIP1-K377R after treatment with 100 nM SMAC mimetic (S) and 10 µM zVAD (Z) for one hour prior to stimulation with 10 ng/ml TNF (T). Cell death was measured after 24 hours as described; the mean percentage of Annexin V staining cells and STD of 3 independent experiments is shown, * denotes p<0.01. All cell lines in Figure 3 express NEMO.

This hypothesis predicts that the non-degradative ubiquitination of RIP1 on lysine 377, which is required for NEMO-binding, should inhibit programmed necrosis. To test this prediction, we reconstituted RIP1-null Jurkat T cells (clone 35.3.13) [5] with wild-type RIP1 or the RIP1-K377R mutant that cannot be ubiquitinated [15] and then compared the sensitivity of these cell lines to programmed necrosis. Consistent with our hypothesis, RIP1-K377R cells undergo cell death when stimulated with TNF in the presence of caspase inhibitor whereas there is no induction of programmed necrosis in RIP1-deficient or RIP1-WT cells, demonstrating that the non-degradative ubiquitination of lysine 377 normally suppresses programmed necrosis (Figure 3A). Subsequent stable transfection of the three cell lines with the IκBαSR reveals that the increased sensitivity of RIP1-K377R cells to programmed necrosis is not simply a result of impaired activation of NFκB. RIP-WT/IκBαSR cells undergo some programmed necrosis, but not to the same extent as RIP1-K377R/IκBαSR cells (Figure S5A). Therefore, non-degradative ubiquitination of lysine 377 of RIP1 prevents programmed necrosis and this protection from cell death does not entirely depend on activation of NFκB, which is identical to the protective role of NEMO. To provide further evidence that the ubiquitination of RIP1 is a key pro-survival step that blocks programmed necrosis, we examined cell death responses after disrupting the activity of the E3 ligases required for RIP1 ubiquitination. RIP1-deficient and RIP1-WT cells were stably transfected with the IκBαSR and then subsequently transfected with either a control protein (RIP-null/IκBαSR/control and RIP-WT/IκBαSR/control) or a TRAF2 deletion mutant that lacks the E3 ligase RING domain [15], [34], [35] (RIP-null/IκBαSR/TRAF2DN and RIP-WT/IκBαSR/TRAF2DN). We confirmed by western blot that the TRAF2DN functions as a dominant negative: RIP1 recruited to TNFR1 is not ubiquitinated in cells that express the TRAF2DN (Figure 3B). We observe some recruitment of RIP1 to TNFR1 in the absence of ligand, which may reflect some competitive binding between TRAF2 and RIP1 [3], [21] with TNFR1 in the steady state. Upon stimulation with TNF in the presence of caspase blockade, it is clear that the TRAF2DN enhances cell death responses in T cells that express RIP-WT but not in RIP1-null cells (Figure 3D and 3E). We confirmed that programmed necrosis induced by expression of TRAF2DN could be blocked by Necrostatin-1 (Figure S5B). Therefore, TRAF2’s E3 ligase activity prevents RIP1 kinase-dependent programmed necrosis from occurring. In addition to TRAF2, several groups have recently reported that treating cells with SMAC mimetics triggers degradation of cIAP1 and cIAP2 E3 ligases [7], [8], which are also required for the ubiquitination of RIP1. Certain cell types undergo RIP1-dependent programmed necrosis when treated with the SMAC mimetic and zVAD-fmk [31], which is consistent with our finding that ubiquitination prevents programmed necrosis. We confirmed that SMAC mimetic treatment of Jurkat T cells triggers programmed necrosis during caspase blockade. RIP1-WT cells undergo programmed necrosis when treated with the SMAC mimetic and TNF for 24 h in the presence of zVAD-fmk, whereas RIP1-deficient T cells do not die (Figure 3F). SMAC mimetic treatment did not significantly enhance programmed necrosis in cells that express the mutant RIP1-K377R, suggesting that non-degradative ubiquitination of lysine 377 of RIP1 by cIAP1 and cIAP2 is a crucial pro-survival event. Typically, treatment of RIP1-WT reconstituted cells with SMAC mimetic results in a 2.6±0.42 fold increase in necrotic cell death compared to 1.17±0.06 fold increase in necrotic cell death of RIP1-K377R cells (data not shown). Therefore, SMAC mimetic can enhance necrosis with RIP1-WT but has little effect if the ubiquitin acceptor site on RIP1 is already missing. Together, these data indicate a correlation between loss of E3 ligase activity and the induction of RIP1-dependent programmed necrosis, which confirms our direct genetic evidence that the ubiquitin acceptor site lysine 377 of RIP1 prevents RIP1 from engaging cell death pathways.

**Figure 4:**
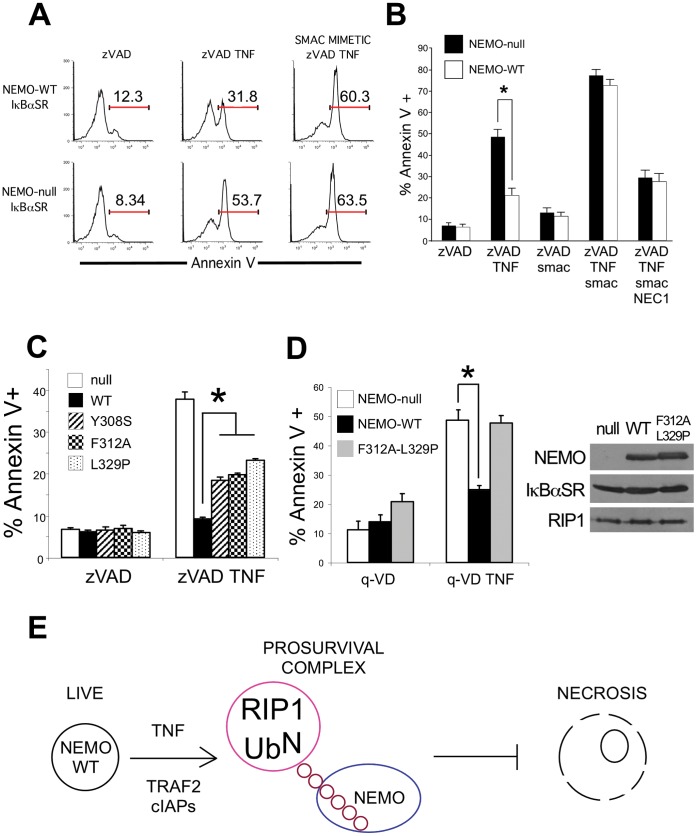
NEMO must be able to bind ubiquitin chains in order to prevent programmed necrosis. (A) NEMO-null cells expressing IκBαSR were transduced with retrovirus encoding a control protein or NEMO-WT. Cells were pre-treated for one hour with 10 µM zVAD with or without 100 nM SMAC mimetic and then stimulated with 10 ng/ml TNF for 24 h. Cell death was quantified by Annexin V staining and flow cytometry. The number above the gate indicates the percentage of cells that display Annexin V staining in one set of histograms representative of several experiments. (B) The bar chart shows the mean percentage of Annexin V staining cells and SEM from three independent experiments, in this case cells were co-incubated with 30 µM Necrostatin-1 to confirm that the cell death is RIP1 kinase-dependent programmed necrosis. (C) NEMO-null cells were reconstituted with retrovirus encoding NEMO-WT or the NOA/UBAN point mutants NEMO-Y308S, NEMO-F312A or NEMO-L329P, which are unable to bind ubiquitinated RIP1 upon ligation of TNFR1. The mean percentage and SEM of cells that stain with Annexin V after 24 h of 10 ng/ml TNF treatment in the presence of 10 µM zVAD is shown, * denotes p<0.05. (D) NEMO-null cells expressing the IκBαSR were reconstituted with NEMO-WT or NEMO-F312A-L329P, a double point mutation in the ubiquitin-binding domain of NEMO. The mean percentage and SEM of cells staining with Annexin V is shown for three independent experiments with cells co-incubated with the caspase inhibitor q-VD and 10 ng/ml TNF for 24 h, * denotes p<0.05. The immunoblot demonstrates that equivalent amounts of NEMO-WT and NEMO-F312A-L329P protein are expressed. (E) These data demonstrate that cells preferentially undergo apoptosis when NEMO cannot bind ubiquitinated RIP1 and that caspase inhibition is required for these cells to switch to RIP1 kinase-dependent programmed necrosis.

In the absence of ubiquitinated RIP1 there is no stimulus-specific interaction of NEMO and RIP1 [16]. Our model that NEMO prevents cell death by interacting with ubiquitinated RIP1 predicts that NEMO should be unable to prevent programmed necrosis when RIP1 is not ubiquitinated. To test this prediction, we treated NEMO-null/IκBαSR cells and NEMO-WT/IκBαSR cells with the SMAC mimetic to prevent ubiquitination of RIP1. As predicted, NEMO-null/IκBαSR and NEMO-WT/IκBαSR cells display the same sensitivity to TNF-induced programmed necrosis after treatment with SMAC mimetic (Figure 4A and 4B). We confirmed that all programmed necrosis induced by the SMAC mimetic in these cells was blocked by Necrostatin-1 (Figure 4B). Therefore, if there are no ubiquitin chains on RIP1, for instance in cells treated with a combination of TNF and SMAC mimetic to prevent ubiquitination, NEMO does not prevent necrosis. In contrast, NEMO prevented necrosis when the SMAC mimetic is absent suggesting that the ubiquitination machinery for RIP1 must be intact in order for NEMO to “restrain” RIP1 from triggering programmed necrosis.

To provide direct genetic evidence that the ability of NEMO to bind ubiquitinated proteins and thus interact with RIP1 prevents programmed necrosis, we reconstituted NEMO-null cells with different NEMO substitution mutations in the ubiquitin-binding domain [11], [33] (NEMO-Y308S, NEMO-F312A and NEMO-L329P). Expression of NEMO-WT and the three mutants was confirmed by western blot and all three mutants have been shown by ourselves and other groups [10], [11], [16], [33], [36] to be unable to recognize K63-linked polyubiquitin chains and to display reduced interaction with ubiquitinated RIP1 during TNF signaling. As expected, reconstitution with NEMO-WT inhibited programmed necrosis, but expression of the three ubiquitin-binding NEMO mutants was not sufficient to prevent programmed necrosis as efficiently as NEMO-WT (Figure 4C). Although the three cell lines expressing the NEMO mutants are more sensitive to programmed necrosis than NEMO-WT cells, there is clearly some protective effect of NEMO-Y308S, NEMO-F312A and NEMO-L329P compared to NEMO-null cells. Since these three mutants are not completely defective in their ability to activate NFκB upon TNF stimulation [16], their heightened resistance to programmed necrosis when compared to the NEMO-null cells could be due to their residual NFκB activity. Therefore, we conducted another experiment to confirm that the ubiquitin-binding activity of NEMO was responsible for NEMO’s NFκB-independent pro-survival activity. NEMO-null cells were transfected with a control protein, NEMO-WT or the double substitution mutant NEMO-F312A-L329P and then each cell line was stably transfected with the IκBαSR. Under this experimental condition, whereby there is no longer any NFκB-mediated gene transcription, the NEMO-F312A-L329P/IκBαSR cells now display the same sensitivity to programmed necrosis as NEMO-null/IκBαSR cells (Figure 4D and Figure S6). Therefore, the inability of NEMO to associate with ubiquitinated proteins such as RIP1 correlates with enhanced programmed necrosis indicating that the ubiquitin-binding domain of NEMO is required for NEMO to prevent RIP1 from initiating programmed necrosis. In conclusion, the activity of the E3 ligases that ubiquitinate RIP1 is required for NEMO to prevent programmed necrosis (Figure 4E) and we present direct genetic evidence that the residues that mediate stimulus-specific interaction of ubiquitinated RIP1 and NEMO [11], [16], [33] underpin the ability of NEMO to inhibit RIP1-dependent programmed necrosis. These observations are consistent with our model that NEMO binding to ubiquitinated RIP1 “restrains” RIP1 from engaging the cell death machinery that orchestrates programmed necrosis.

### The Deubiquitinase CYLD is Required for Programmed Necrosis in NEMO-deficient Cells

**Figure 5:**
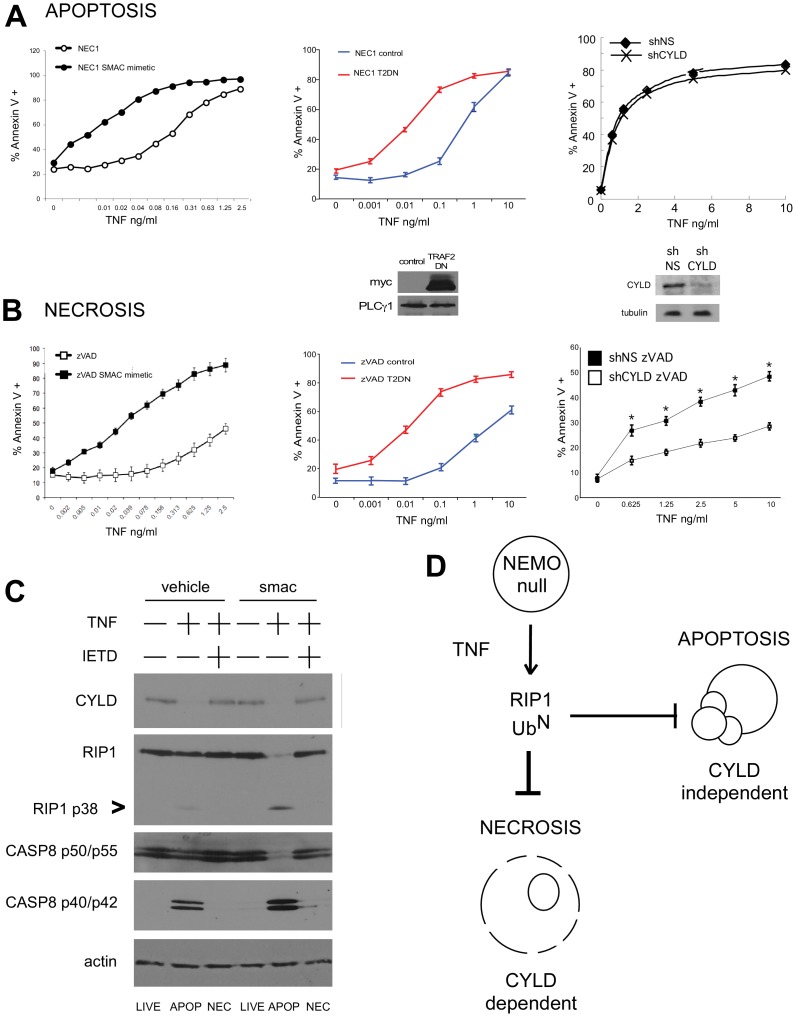
Deubiquitination of RIP1 accelerates both apoptotic and necrotic cell death in NEMO-deficient cells but CYLD is specifically required for programmed necrosis. (A) Apoptosis of NEMO-deficient 8321 cells was examined by experiments conducted in the presence of 30 µM Necrostatin-1 to prevent necrosis. NEMO null cells were pre-treated with 100 nM SMAC mimetic and 10 µM zVAD for one hour, the line graph shows the mean percentage and SEM from three independent experiments of Annexin V staining cells after 24 h treatment with different doses of TNF, * denotes *p*<0.05– (left panel). NEMO-null cells were transduced with either a control protein or the TRAF2DN and co-incubated with different doses of TNF for 24 h. The mean percentage and SEM of Annexin V staining cells is shown for three independent experiments, * denotes *p*<0.05. (middle panel). Immunoblot demonstrates expression of TRAF2DN protein. NEMO-null cells transduced with retrovirus encoding non-targeting (shNS) or CYLD-targeting short hairpins (shCYLD) were co-incubated with different doses of TNF for 24h. Cell death was quantified by Annexin V staining and flow cytometry. The mean and SEM of three independent experiments is shown, * denotes *p*<0.05 (right panel). Immunoblot indicates efficient knockdown of CYLD protein in shCYLD cells. Data shown is representative of similar results obtained with two different CYLD-targeting hairpins. (B) Necrosis was examined by conducting a similar series of experiments to those in (A) in the presence of 10 µM zVAD. (C) NEMO-null cells were treated with 100 nM SMAC mimetic in the absence or presence of 100 µM of caspase 8-specific inhibitor IETD and then stimulated with 10 ng/ml TNF. Lysates were blotted with antibody specific for CYLD, RIP1, Caspase 8 and actin as a loading control. The known caspase cleavage product of RIP1 is indicated with an >. The form of cell death that corresponds to the stimuli in each lane is denoted at the bottom of the immunoblot: APOP  =  apoptosis, NEC  =  necrosis. (D) Model summarizing cell death data: ubiquitination of RIP1 blocks both apoptosis and necrosis in NEMO-null cells but the deubiquitinase CYLD is specifically required for programmed necrosis to occur.

It has been previously reported that RIP1 in the pro-necrotic complex does not display the “laddering” characteristic of polyubiquitination [30]. In contrast, RIP1 is clearly polyubiquitinated when recruited to TNFR1 (Complex I) and in the pro-apoptotic Complex II [37]. These earlier studies together with the observations in Figure 3 and 4 that inhibiting RIP1 ubiquitination favors programmed necrosis suggest the possibility that it is non-ubiquitinated RIP1 that activates the necrotic machinery. In NEMO-deficient cells, non-degradative ubiquitination of RIP1 still occurs because the E3 ligases are still active, therefore, if non-ubiquitinated RIP1 forms the active pro-necrotic complex we hypothesized that blocking the activity of the E3 ligases that ubiquitinate RIP1 should further enhance cell death in NEMO-deficient cells. NEMO-deficient cells were treated with Necrostatin-1 (to ensure all cell death is apoptosis) or zVAD-fmk (to ensure all cell death is necrosis) and different doses of TNF for 24 h in the presence of SMAC mimetic. NEMO-deficient T cells treated with SMAC mimetic are more sensitive to either TNF-triggered apoptosis or programmed necrosis (Figure 5A, left panel and 5B, left panel), which supports our hypothesis that it is non-ubiquitinated RIP1 that mediates cell death. To further confirm this finding, NEMO-deficient T cells were stably transfected with either a control protein or the TRAF2DN. After 24 hours stimulation with TNF in the presence of Necrostatin-1 or zVAD-fmk (i.e. all cell death is apoptotic or necrotic, respectively), we find that NEMO-null/TRAF2DN cells are more sensitive to apoptosis and programmed necrosis than NEMO-null/control cells (Figure 5A, middle panel and 5B, middle panel). Together, these observations suggest that non-ubiquitinated RIP1 is more able to activate both the apoptotic and necrotic machinery, which suggests the possibility that in NEMO-deficient cells the non-degradative ubiquitin chains on RIP1 must be removed before these cells enter cell death pathways.

The deubiquitinase CYLD, which has been reported to remove non-degradative ubiquitin chains from RIP1 during spermatogenesis [38], was identified as an essential gene for programmed necrosis to occur [39]. We hypothesized that CYLD might function downstream of NEMO and contribute to programmed necrosis in NEMO-deficient cells by removing ubiquitin chains from RIP1. To test this hypothesis, we first confirmed that CYLD is able to remove K63-linked polyubiquitin chains from RIP1. We find that CYLD can interact with RIP1 in HEK 293 cells upon co-transfection (Figure S7A) and that CYLD is an efficient deubiquitinase for RIP1 (Figure S7B). HEK 293 cells were transfected with RIP1 and a ubiquitin mutant that can only form K63-linked chains. Immunoprecipitation of the RIP1 from these cells and western blotting for HA-tagged ubiquitin confirms that RIP1 can be conjugated to K63-linked polyubiquitin chains. However, co-transfection of CYLD prevented the K63-linked polyubiquitination of RIP1 (Figure S7B). We then tested to see if CYLD is required for apoptosis or programmed necrosis in the NEMO-deficient cells by knocking down the endogenous CYLD protein with a retroviral vector encoding a hairpin targeting CYLD. Knockdown of CYLD had no effect on cell death of NEMO-deficient cells treated with TNF in the absence of zVAD, i.e. CYLD is not required when the cell death is apoptotic (Figure 5A, right panel). In contrast, knockdown of CYLD inhibited programmed necrosis of NEMO-deficient cells stimulated with different doses of TNF for 20 h in the presence of zVAD (Figure 5B, right panel), confirming that CYLD is required for programmed necrosis. Knockdown of CYLD had no effect on the ability of NEMO-deficient T cells to undergo apoptosis; NEMO-null/shNS and NEMO-null/shCYLD are equally sensitive to the induction of apoptosis over 20 h (Figure 5A, right panel), despite the fact that blocking RIP1 ubiquitination enhances apoptosis in NEMO-null cells (Figure 5A left and middle panel). This observation that CYLD is not required for apoptosis is not too surprisingly in light of our recent finding that CYLD is a substrate for Caspase 8 and is rapidly removed by the Caspase 8 activated during apoptosis [40]. In Figure 5C, we confirm by western blot that full-length CYLD protein is lost from NEMO-deficient cells stimulated with TNF; therefore CYLD is not present during apoptosis. Blockade of Caspase 8 activity with the pharmacological inhibitor IETD prevents the loss of full-length CYLD and this correlates with the entry of the NEMO-deficient cells into cell death by programmed necrosis. In the absence of NEMO, ubiquitination of RIP1 prevents both apoptotic and necrotic cell death, but programmed necrosis specifically requires stabilization of CYLD and presumably deubiquitination of RIP1, as summarized in the cartoon in Figure 5D. Therefore, we conclude that CYLD is required for programmed necrosis to occur in NEMO-deficient cells most likely because it is needed to remove polyubiquitin from RIP1 in order for RIP1 to engage the necrotic pathway. Overall, our data demonstrates that disruption of the first cell death checkpoint in TNF signaling leads to a necrotic response if the apoptotic machinery is inhibited.

## Discussion

TNFR1 orchestrates the formation of pro-survival and potentially pro-death signaling complexes, often within the same cell. However, the dominant outcome of TNF signaling is cell survival, which suggests that potent cytoprotective mechanisms exist to keep the death complexes from triggering aberrant deletion of important cells [41]. It is critical for cells to avoid a “vile forfeit of untimely death” by programmed necrosis because this type of cell death can be extremely pro-inflammatory [21], [30], as exemplified by the tissue damage seen in ischemia-reperfusion injuries [29]. We have recently proposed that there are two major checkpoints in the induction of cell death by TNF that are controlled by RIP1 [17]. After ligation of TNFR1, RIP1 is rapidly conjugated to non-degradative ubiquitin chains by the concerted action of the E3 ligases TRAF2, cIAP1 and cIAP2 [7], [9]. Ubiquitinated RIP1 is less able to bind the cell death mediator CASPASE 8 and thus ubiquitination of RIP1 prevents apoptosis [15]. One signaling event that makes a major contribution to this cytoprotective effect of RIP1 ubiquitination is recruitment of the adaptor protein NEMO. NEMO binding restrains RIP1 from activating apoptosis at early time-points; this survival-promoting activity is effective before transcription-dependent cytoprotective mechanisms are evident [16]. At later time-points, the activation of NFκB mediated by the interaction of NEMO and RIP1 further augments this early pro-survival activity, presumably by increasing expression of NFκB-dependent pro-survival factors such as cFLIP.

**Figure 6:**
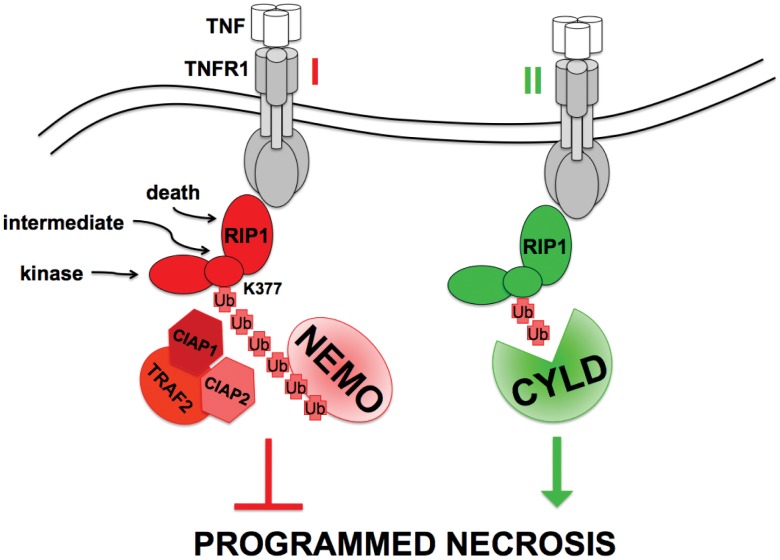
Recruitment of NEMO to ubiquitinated RIP1 prevents programmed necrosis. A model that represents the early pro-survival effect of RIP1 ubiquitination is shown. In the first few minutes after ligation of TNFR1, lysine 377 of RIP1 is conjugated to non-degradative ubiquitin chains by the concerted action of the E3 ligases cIAP1, cIAP2 and TRAF2. The bi-partite ubiquitin binding domain of NEMO docks with ubiquitinated RIP1 and this stimulus-specific interaction restrains the kinase domain of RIP1 from instigating cell death by programmed necrosis. In the absence of NEMO, the ubiquitin chains on RIP1 are likely removed by the action of the CYLD deubiquitinase and RIP1 becomes a pro-death signaling molecule that can trigger programmed necrosis when caspase activity is blocked. The pro-survival complex of TNFR1 (I) is the default scenario but a pro-death complex (II) is predicted to arise physiologically when the ubiquitination of RIP1 is prevented by ligation of TNFR2, which triggers degradation of TRAF2, cIAP1 and cIAP2.

In this study we report that the ubiquitination of RIP1 and binding of NEMO effectively prevents RIP1 from driving cell death by programmed necrosis when caspase activity is blocked. Disruption of the ubiquitination of RIP1 by either mutation of the acceptor lysine or inactivation of the TRAF2, cIAP1 and cIAP2 E3 ligases predisposes cells to undergo programmed necrosis when treated with TNF in the presence of caspase inhibitors. Similarly, cells harboring NEMO mutations that disrupt the association with ubiquitinated RIP1 also exhibit greater sensitivity to programmed necrosis. Thus binding of NEMO to ubiquitinated RIP1 is a critical early event that prevents programmed necrosis (Figure 6). While this early survival signal is independent of NFκB, subsequent induction of pro-survival genes by NFκB leads to a more sustained protection from necrosis. There are several possible mechanisms by which NEMO may restrain RIP1 from engaging the necrotic machinery. The formation of a complex of ubiquitinated RIP1 and NEMO may prevent RIP1 from binding other components of the cell death machinery, such as RIP3 [30], [31], [32], via a variety of possible mechanisms such as altering the subcellular localization of RIP1 complexes or direct steric hindrance of RIP3 binding. The interaction of NEMO with RIP1 may activate kinase complexes that prevent RIP1 or other signaling molecules from functioning in the necrotic apparatus. For example, NEMO is required for IKKβ and IKKα to phosphorylate and inhibit the activity of the deubiquitinase CYLD [42], a critical component of the necrotic machinery. Indeed, IKKβ knockout MEFs undergo caspase-independent cell death when stimulated with TNF whereas cell death in IKKα knockout MEFs is restricted to apoptosis [43], suggesting very specific regulation of different forms of cell death by the kinases activated by NEMO. Recruitment and activation of Tak1 in the TNFR1 complex requires ubiquitination of RIP1 [11], [12]; recruited Tak1 then phosphorylates and activates the IKK complex. It has been shown recently that Tak1 activity can also prevent RIP1 from forming a necrosome [44], [45]. In NEMO deficient cells it is clear that blocking the activity of the E3 enzymes TRAF2, cIAP1 and cIAP2 accelerates necrosis, whereas removing the deubiquitinase CYLD impedes necrosis. This suggests that ubiquitination of RIP1 has cytoprotective functions mediated by events in addition to the recruitment of NEMO. There are several other ubiquitin-binding proteins that interact with RIP1, for example ABIN1 [46], p62 [47], [48] and TAB2/TAB3 [12], [49]. Cells from knockout mice that lack these ubiquitin binding proteins, or the downstream signaling molecules they recruit to RIP1 such PKCς or TAK1, are hypersensitive to TNF-mediated cell death [46], [50], [51], therefore, we speculate that these ubiquitin-binding proteins may also prevent ubiquitinated RIP1 from triggering cell death in an NFκB-independent manner. Our data demonstrates that the prevention of RIP1 ubiquitination in NEMO-null cells can accelerate both apoptosis (Figure 5A) and necrosis (Figure 5B) but CYLD is only required for necrosis and not apoptosis of NEMO-null cells. Indeed, processing of CYLD by Caspase 8 removes CYLD to prevent necrosis suggesting that in apoptotic cells CYLD is not present and therefore unlikely to play a major role in TNF-mediated apoptosis. This raises the possibility that another deubiquitinase enzyme might be important for enabling RIP1 to engage CASPASE 8 and the apoptotic machinery, whereas CYLD is crucial for RIP1 to engage the necrosis machinery. The RIP1 deubiquitinase USP2a may be a good candidate as it can promote apoptosis in Hela cells treated with TNF and cycloheximide suggesting that this enzyme promotes apoptosis independent of its effects on the NFκB pathway [52]. Alternatively, we speculate that the prevention of RIP1 ubiquitination accelerates apoptosis by loss of Complex I formation but that the specific removal of these ubiquitin chajns is not a pre-requisite for RIP1 to bind Caspase 8 to initiate apoptosis when components of Complex I, such as NEMO, are already absent. To summarize our findings, binding of NEMO to ubiquitinated RIP1 restrains RIP1 from initiating cell death by programmed necrosis. We conclude that in the absence of NEMO and when Caspase 8 is inhibited from cleaving CYLD, RIP1 is deubiquitinated and can interact with and activate downstream necrosis signaling molecules such as RIP3.

Cell survival is likely to be the dominant response during TNF stimulation because this is required for cells to subsequently activate gene expression programs that control a multitude of inflammatory and immune processes [41]. However, it is also important that TNF can rapidly trigger cell death when appropriate. The ability of RIP1 ubiquitination and NEMO binding to prevent cell death early after receptor ligation presents the ideal opportunity for other signals to regulate this pro-survival switch to trigger cell death when necessary. This is epitomized by the ability of TNFR2 to enhance cell death signals transmitted via TNFR1 [53]. Ligation of TNFR2 triggers the degradation of the E3 ligases TRAF2, cIAP1 and cIAP2 [54], [55]: an environment that would leave RIP1 unmodified by ubiquitin chains and thus free to engage cell death machinery. The necessity for this rapidly inducible cell death response is clear in the context of infection with viruses that encode potent inhibitors of caspases such as Vaccinia. TNFR2 ligation enables cells infected with vaccinia virus to die by programmed necrosis and trigger an inflammatory response that is essential for the clearance of the virus [21], [30].

NEMO is a critical component of signaling complexes that mediate immune functions, most notably the kinases that activate the NFκB and IRF3 transcription factors [56]. We speculate that regulation of NEMO protein levels or function would thus be an attractive target for pathogens to evade the immune response. In support of this idea, Shigella encodes an E3 ligase that triggers degradation of NEMO and inhibits NFκB-dependent immune functions [57]. Based on our studies, this loss of NEMO during infection would be predicted to leave infected cells vulnerable to the induction of programmed necrosis by TNF. The possibility that programmed necrosis might be engaged in physiological situations where NEMO is targeted by pathogens is supported by the fact that Shigella-infected epithelial cells can undergo a necrotic type death [58], [59]. Similarly, the M45 protein of murine cytomegalovirus also targets NEMO for degradation to prevent cytokine production [60]. We would predict that M45-driven loss of NEMO would predispose MCMV-infected cells to cell death by programmed necrosis, however, the MCMV M45 also contains a RIP homology interaction motif (RHIM) that prevents programmed necrosis by disrupting the interaction between RIP1 and RIP3 [61]. Since the ability of the MCMV M45 protein to trigger degradation of NEMO in autophagosomes is independent of the RHIM domain [60], we speculate that the M45 protein may have co-evolved the ability to block programmed necrosis in order to survive the loss of NEMO. It will be interesting to investigate if loss of NEMO occurs in other infections and whether or not programmed necrosis is utilized in this context to trigger both cell death of infected cells and initiate a pro-inflammatory response to circumvent the pathogen immune evasion strategy.

The NFκB-independent ability of NEMO to prevent cell death by apoptosis and necrosis may have important implications for understanding the spectrum of developmental defects, inflammatory disease and immune deficiencies associated with NEMO mutations in humans. For example, hypersensitivity to TNF-induced cell death does not necessarily correlate with impaired activation of NFκB by different NEMO mutants [62]. In this context, alteration in the ability of NEMO to regulate inflammatory and immunogenic cell death by different mutations in NEMO could determine whether a patient displays autoimmunity.

In conclusion, cells can toggle between cell survival and cell death pathways when stimulated with TNF by modulating the ubiquitination status of RIP1. Disruption of the early pro-survival checkpoint in the TNFR1 pathway mediated by NEMO binding to RIP1 is a very efficient trigger of cell death, both by apoptosis and programmed necrosis. Therefore, this report details a major molecular mechanism initiated by TNFR1 that prevents necrotic cell death and provides a rationale for understanding the ability of TNFR2 or SMAC mimetics to trigger necrosis. NEMO and RIP1 are key signaling mediators that regulate survival and death responses in diverse signaling events such as TCR activation [63], [64] and DNA damage [65] in addition to this study of TNFR1-mediated cell death. Therefore, a more detailed understanding of the additional signaling events that control this switch should provide novel approaches for therapeutic intervention in RIP1 or TNF-driven pathologies such as chronic inflammatory diseases, ischemia-reperfusion injuries and cancer.

## Materials and Methods

### Cell Lines

Parental Jurkat clone SVT35 and its derivative RIP1-null clone 35.3.13 have been previously described [5]. RIP1-null cells [5] were transduced with retrovirus encoding either a control protein (bacterial glutathione transferase), RIP1-WT, RIP1-K377R, IκBαSR and the TRAF2DN as described before [15]. Parental Jurkat clone 3T8 and its derivative NEMO-null clone 8321 have been previously described [66]. NEMO-null T cells [66] were transduced with retrovirus encoding NEMO-WT or point mutations in the ubiquitin binding domain generated by site directed mutagenesis (Stratagene Quikchange XL) as described previously [16].

### Reagents and Antibodies

Phycoerythrin conjugated Annexin V (Pharmingen), propidum iodide, Necrostatin-1 and zVAD-fmk (Calbiochem), q-VD-OPH (SMBiochemicals), recombinant human TNF (R&D), specific antibodies used: Caspase 3 and PARP (Cell Signaling), NEMO and tubulin (Santa Cruz), Thy1 (Cedarlane Laboratories), Fas (Upstate), HA and myc (Roche), RIP1 (Pharmingen), FLAG antibody and beads (Sigma), TNFR1 (R&D). The CYLD-specific antibody [42] and SMAC mimetics [31] were generously provided by Drs. Shao Cong Sun (MD Anderson Cancer Center, Houston, TX) and Dr. Xiaodong Wang (UT Southwestern, Dallas, TX), respectively.

### Immunoprecipitation

5×10^7^ T cells stimulated with 100 ng/ml TNF for 5 minutes were lysed in NP-40 lysis buffer and incubated overnight with TNFR1-specific antibody and isolated with protein A beads as described previously (He et al., 2002). To detect RIP1 ubiquitination upon overexpression in HEK 293 cells, triton-soluble lysates were denatured by boiling in 1% SDS for 5 minutes and then subjected to FLAG-specific immunoprecipitation as described previously [67].

### Annexin V Staining

Approximately 5×10^5^ T cells per sample were stimulated as described and then washed once with Annexin V binding buffer (10 mM HEPES, 140 mM NaCl, 2.5 mM CaCl_2,_ pH7.5), resuspended in binding buffer and incubated with 1 mg/L Annexin V-PE for 5 to 15 minutes before immediate analysis on a Becton Dickinson FACScan flow cytometer.

### DNA Fragmentation Assay

Jurkat cells were washed with PBS and then lysed for 30 minutes on ice (20 mM Tris-HCl, 4 mM EDTA, 0.4% Triton-X 100 pH7.4). Protein in the cell lysate was removed by mixing with an equal volume of phenol/chloroform pH 8.0. DNA fragments were precipitated from the aqueous phase by ethanol precipitation and electrophoresed in a 1.2% agarose gel. Separated DNA fragments were visualized by Ethidum bromide staining.

### Stable Knockdown

T cells were transduced with pSUPERretro-GFP or pSUPERretro-puromycin encoding non-specific or CYLD [67] or RIP1 targeting short hairpins as described previously [16].

## Supporting Information

Figure S1 **TNF induces caspase-independent cell death in NEMO-deficient T cells.** (A) 3T8 parental cells transduced with the IκBαSR and 8321 NEMO-null cells were pre-treated with the indicated doses of zVAD-fmk for one hour and then stimulated with 10 ng/ml TNF or 100 ng/ml anti-FAS for 20 h. Cell death was quantified by Annexin V staining and flow cytometry. The percentage of cells that stain with Annexin V is shown. (B) The 3T8/IκBαSR and 8321 NEMO-null cells were pre-treated with zVAD-fmk and stimulated with TNF or anti-FAS as described in (A) and the cell viability was quantified using the Cell Titer-96 Aqueous One Solution cell proliferation assay (Promega). The mean viability and standard deviation is shown for triplicate cultures. These graphs indicate that NEMO-null cells undergo programmed necrosis when stimulated with TNF in the presence of caspase inhibitors, whereas FAS-mediated cell death in both 3T8/IκBαSR and 8321 NEMO-null cells is entirely caspase-dependent.
(TIF)

Figure S2 **NFκB-mediated gene transcription is absent in IκBαSR-transfected cells.** (A) The 8321 NEMO-null cells contain a heterologous NFκB-dependent Thy1 reporter gene. The three cell lines described in Figure 1B (NEMO-null, NEMO-WT clone A and B) were stimulated with TNF and stained with antibody specific for Thy1 and analyzed by flow cytometry to confirm that NEMO reconstitution in clones A and B resulted in a reconstitution of the NFκB pathway. As expected, NEMO-null cells express no Thy1 upon TNF stimulation whereas NEMO-WT clone A and B cells activate NFκB and express Thy1 (top three panels). The three cell lines were subsequently transduced with retrovirus encoding the IκBαSR to block all NFκB-mediated gene transcription. The three cell lines (NEMO-null/IκBαSR and NEMO-WT/IκBαSR clones A and B) do not express Thy1 after TNF stimulation (bottom three panels) indicating that there is no NFκB-dependent gene transcription in these cells. (B) Time-course of caspase-independent cell death in NEMO-deficient and NEMO-WT reconstituted 8321 cells treated with three doses of zVAD-fmk (2.5, 5 and 10 µM) and 10 ng/ml TNF. The mean and SEM of three independent experiments is shown.
(TIF)

Figure S3 **Multiple cell death assays indicate that NEMO-null cells die in response to TNF in the presence of zVAD.** (A) 8321 NEMO-null cells and the parental 3T8 cells transduced with the IκBαSR were pre-treated with zVAD for one hour and then stimulated with the indicated doses of TNF for 20 hours. The cell viability was measured using the Cell Titer-96 Aqueous One Solution cell proliferation assay (Promega) and mean values ± standard deviation are shown from triplicate cultures. (B) The NEMO-null/IκBαSR and NEMO-WT/IκBαSR clone A and B cell lines were pre-treated with zVAD and stimulated with the indicated doses of TNF for 20 h, cell death was measured by LDH release assay (Roche). The mean LDH release values ± standard deviation are shown for triplicate cultures. (C) The NEMO-null/IκBαSR and NEMO-WT/IκBαSR clone A and B cell lines were pre-treated with zVAD and stimulated with the indicated doses of TNF for 20 h, cell death was measured by staining cells with propidium iodide and flow cytometry. The percentage of cells that take-up propidium iodide i.e. that have a permeabilised plasma membrane due to cell death is shown. In addition to Annexin V staining, three additional cell death assays shown here confirm that NEMO-null cells undergo cell death in the presence of caspase inhibitors. The caspase-independent cell death in NEMO-deficient cells is not simply due to a lack of NFκB-mediated gene transcription, as parental cells transfected with IκBαSR do not exhibit much caspase-independent cell death (A). (B) and (C) confirm that the presence or absence of NEMO determines whether caspase-independent cell death occurs, independent of NEMO’s role in activating NFκB.
(TIF)

Figure S4 **RIP3 is required for programmed necrosis of NEMO-deficient cells.** 8321 NEMO-deficient cells were transduced with lentivirus encoding two different hairpins targeting RIP3 (Sigma) and then stimulated with 10 ng/ml TNF in the presence of 100 µM zVAD for 24 hours. Cell death was quantified by Annexin V staining and flow cytometry. The mean percentage of cells that are Annexin V + and the standard deviation is shown for one experiment with triplicate samples and is representative of two similar experiments. The immunoblot confirms detectable knockdown with the shRIP3-4 lentivirus but not with the shRIP3-2 lentivirus, consistent with the inhibition of necrosis in shRIP3-4 knockdown but not shRIP3-2 knockdown cells.
(TIF)

Figure S5 **Ubiquitination of lysine 377 of RIP1 prevents programmed necrosis.** (A) RIP1-null Jurkat T cells were transduced with either a control protein, RIP1-WT or RIP1-K377R (left panel). Cells were pre-treated for one hour with 10 µM zVAD and then stimulated with the indicated doses of TNF for 24 hours. Cell death was quantified by Annexin V staining and flow cytometry. RIP1-null cells reconstituted with RIP1-WT or RIP1-K377R were subsequently transduced with the IκBαSR and caspase-independent cell death was measured after 24 hours of stimulation with TNF (right panel). (B) RIP1-null Jurkat T cells expressing IκBαSR were transduced with either a control protein or RIP1-WT and then subsequently with a control protein or the TRAF2DN. The bar chart displays the mean percentage and SEM from three independent experiments of propidium iodide staining of cells after 24 h of culture in vehicle, zVAD, Necrostatin-1 or a combination of zVAD and Necrostatin-1 either in the presence or absence of 10 ng/ml TNF for 24 h, * denotes p<0.05. The data demonstrates that TRAF2DN conferred sensitivity to caspase-independent cells only when RIP1 is present and this is caspase-independent cell death is blocked by Necrostatin-1.
(TIF)

Figure S6 **Ubiquitin recognition by NEMO prevents programmed necrosis.** NEMO-deficient Jurkats T cells expressing IκBαSR were transduced with either a control protein, NEMO-WT or the ubiquitin-binding deficient mutant NEMO-F312A-L329P. The bar chart displays the mean percentage and SEM from three independent experiments of Annexin V staining cells after 24 h of culture in vehicle, 10 µM zVAD, 30 µM Necrostatin-1 or a combination of zVAD and Necrostatin-1 either in the presence or absence of 10 ng/ml TNF for 24 h, * denotes p<0.05. The data demonstrates that NEMO-deficient and NEMO-F312A-L329P cells preferentially undergo apoptosis and caspase blockade is required for entry into RIP1 kinase-dependent programmed necrosis.
(TIF)

Figure S7 **CYLD binds RIP1 and reduces RIP1 ubiquitination.** (A) HEK 293 cells were transfected with myc-RIP1 together with FLAG-CYLD, GST or FLAG-p100, with the latter two as negative controls. FLAG-CYLD and FLAG-p100 were immunoprecipitated from triton-soluble lysates and immunoblotted with myc and FLAG-specific antibodies. A sample of lysate was blotted for myc and FLAG to show equivalent expression of each construct. (B) HEK 293 cells were transfected with HA-ubiquitin that contains only lysine 63, FLAG-RIP1 and myc-CYLD. FLAG-RIP1 was immunoprecipitated after SDS-denaturation of triton-soluble lysates and blotted with antibody specific for HA to detect ubiqutinated RIP1. A sample of the lysate was blotted for HA, FLAG and myc to detect transfected ubiquitin, RIP1 and CYLD, respectively.
(TIF)
