# Supplementary figures and images for: NEMO Inhibits Programmed Necrosis in an NFκB-Independent Manner by Restraining RIP1
Source: PLoS One. 2012 Jul 26;7(7):e41238. doi: 10.1371/journal.pone.0041238 (PMC3406058; doi:10.1371/journal.pone.0041238)

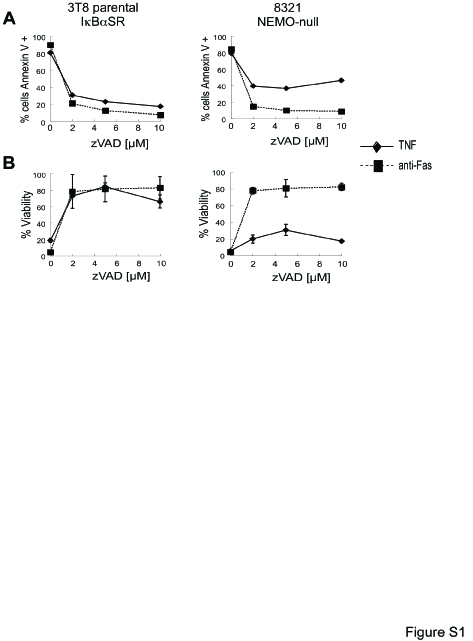

Supplement: Figure S1 — TNF induces caspase-independent cell death in NEMO-deficient T cells. (A) 3T8 parental cells transduced with the IκBαSR and 8321 NEMO-null cells were pre-treated with the indicated doses of zVAD-fmk for one hour and then stimulated with 10 ng/ml TNF or 100 ng/ml anti-FAS for 20 h. Cell death was quantified by Annexin V staining and flow cytometry. The percentage of cells that stain with Annexin V is shown. (B) The 3T8/IκBαSR and 8321 NEMO-null cells were pre-treated with zVAD-fmk and stimulated with TNF or anti-FAS as described in (A) and the cell viability was quantified using the Cell Titer-96 Aqueous One Solution cell proliferation assay (Promega). The mean viability and standard deviation is shown for triplicate cultures. These graphs indicate that NEMO-null cells undergo programmed necrosis when stimulated with TNF in the presence of caspase inhibitors, whereas FAS-mediated cell death in both 3T8/IκBαSR and 8321 NEMO-null cells is entirely caspase-dependent. (TIF) [file pone.0041238.s001.tif]

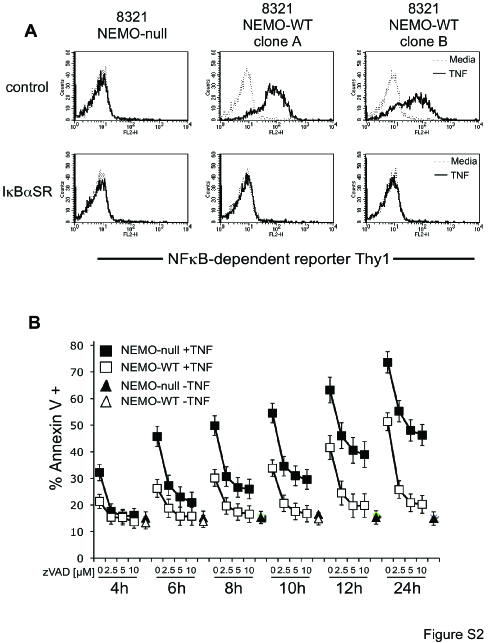

Supplement: Figure S2 — NFκB-mediated gene transcription is absent in IκBαSR-transfected cells. (A) The 8321 NEMO-null cells contain a heterologous NFκB-dependent Thy1 reporter gene. The three cell lines described in Figure 1B (NEMO-null, NEMO-WT clone A and B) were stimulated with TNF and stained with antibody specific for Thy1 and analyzed by flow cytometry to confirm that NEMO reconstitution in clones A and B resulted in a reconstitution of the NFκB pathway. As expected, NEMO-null cells express no Thy1 upon TNF stimulation whereas NEMO-WT clone A and B cells activate NFκB and express Thy1 (top three panels). The three cell lines were subsequently transduced with retrovirus encoding the IκBαSR to block all NFκB-mediated gene transcription. The three cell lines (NEMO-null/IκBαSR and NEMO-WT/IκBαSR clones A and B) do not express Thy1 after TNF stimulation (bottom three panels) indicating that there is no NFκB-dependent gene transcription in these cells. (B) Time-course of caspase-independent cell death in NEMO-deficient and NEMO-WT reconstituted 8321 cells treated with three doses of zVAD-fmk (2.5, 5 and 10 µM) and 10 ng/ml TNF. The mean and SEM of three independent experiments is shown. (TIF) [file pone.0041238.s002.tif]

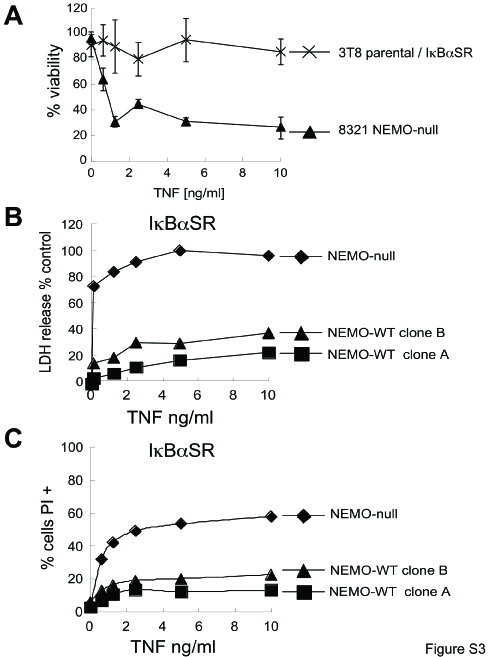

Supplement: Figure S3 — Multiple cell death assays indicate that NEMO-null cells die in response to TNF in the presence of zVAD. (A) 8321 NEMO-null cells and the parental 3T8 cells transduced with the IκBαSR were pre-treated with zVAD for one hour and then stimulated with the indicated doses of TNF for 20 hours. The cell viability was measured using the Cell Titer-96 Aqueous One Solution cell proliferation assay (Promega) and mean values ± standard deviation are shown from triplicate cultures. (B) The NEMO-null/IκBαSR and NEMO-WT/IκBαSR clone A and B cell lines were pre-treated with zVAD and stimulated with the indicated doses of TNF for 20 h, cell death was measured by LDH release assay (Roche). The mean LDH release values ± standard deviation are shown for triplicate cultures. (C) The NEMO-null/IκBαSR and NEMO-WT/IκBαSR clone A and B cell lines were pre-treated with zVAD and stimulated with the indicated doses of TNF for 20 h, cell death was measured by staining cells with propidium iodide and flow cytometry. The percentage of cells that take-up propidium iodide i.e. that have a permeabilised plasma membrane due to cell death is shown. In addition to Annexin V staining, three additional cell death assays shown here confirm that NEMO-null cells undergo cell death in the presence of caspase inhibitors. The caspase-independent cell death in NEMO-deficient cells is not simply due to a lack of NFκB-mediated gene transcription, as parental cells transfected with IκBαSR do not exhibit much caspase-independent cell death (A). (B) and (C) confirm that the presence or absence of NEMO determines whether caspase-independent cell death occurs, independent of NEMO’s role in activating NFκB. (TIF) [file pone.0041238.s003.tif]

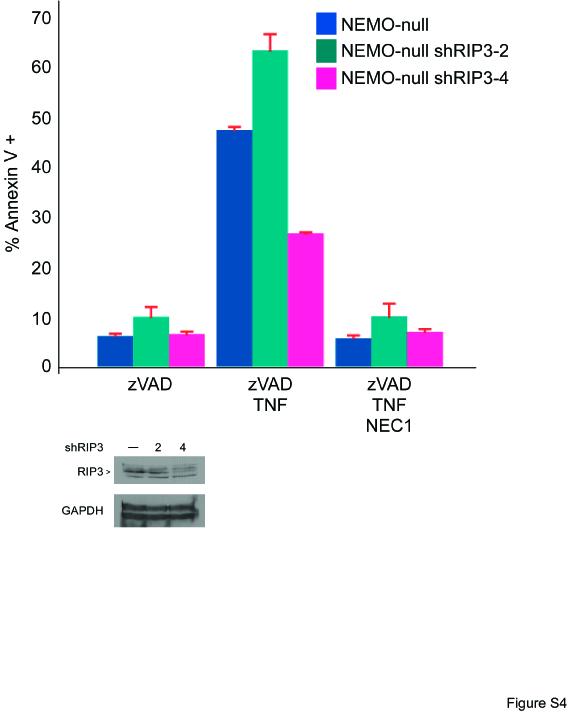

Supplement: Figure S4 — RIP3 is required for programmed necrosis of NEMO-deficient cells. 8321 NEMO-deficient cells were transduced with lentivirus encoding two different hairpins targeting RIP3 (Sigma) and then stimulated with 10 ng/ml TNF in the presence of 100 µM zVAD for 24 hours. Cell death was quantified by Annexin V staining and flow cytometry. The mean percentage of cells that are Annexin V + and the standard deviation is shown for one experiment with triplicate samples and is representative of two similar experiments. The immunoblot confirms detectable knockdown with the shRIP3-4 lentivirus but not with the shRIP3-2 lentivirus, consistent with the inhibition of necrosis in shRIP3-4 knockdown but not shRIP3-2 knockdown cells. (TIF) [file pone.0041238.s004.tif]

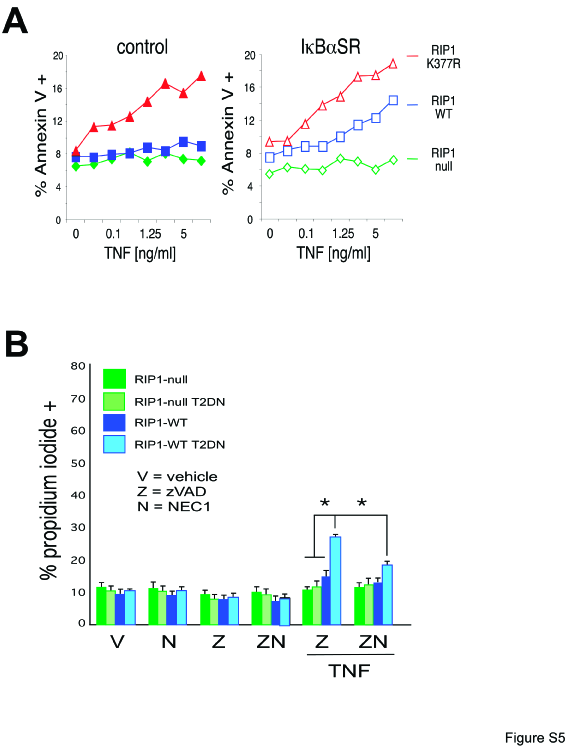

Supplement: Figure S5 — Ubiquitination of lysine 377 of RIP1 prevents programmed necrosis. (A) RIP1-null Jurkat T cells were transduced with either a control protein, RIP1-WT or RIP1-K377R (left panel). Cells were pre-treated for one hour with 10 µM zVAD and then stimulated with the indicated doses of TNF for 24 hours. Cell death was quantified by Annexin V staining and flow cytometry. RIP1-null cells reconstituted with RIP1-WT or RIP1-K377R were subsequently transduced with the IκBαSR and caspase-independent cell death was measured after 24 hours of stimulation with TNF (right panel). (B) RIP1-null Jurkat T cells expressing IκBαSR were transduced with either a control protein or RIP1-WT and then subsequently with a control protein or the TRAF2DN. The bar chart displays the mean percentage and SEM from three independent experiments of propidium iodide staining of cells after 24 h of culture in vehicle, zVAD, Necrostatin-1 or a combination of zVAD and Necrostatin-1 either in the presence or absence of 10 ng/ml TNF for 24 h, * denotes p<0.05. The data demonstrates that TRAF2DN conferred sensitivity to caspase-independent cells only when RIP1 is present and this is caspase-independent cell death is blocked by Necrostatin-1. (TIF) [file pone.0041238.s005.tif]

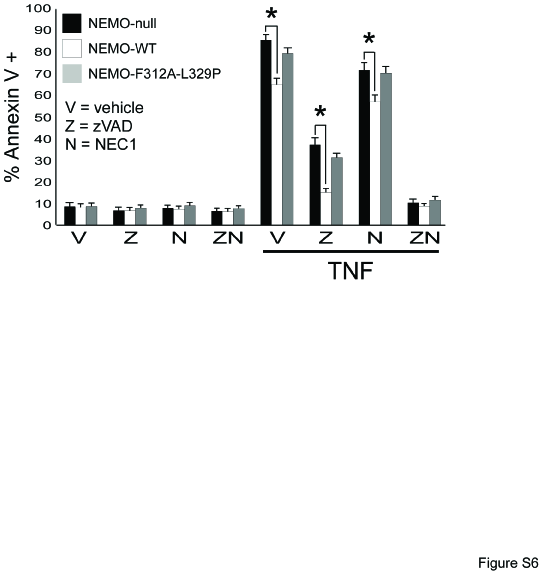

Supplement: Figure S6 — Ubiquitin recognition by NEMO prevents programmed necrosis. NEMO-deficient Jurkats T cells expressing IκBαSR were transduced with either a control protein, NEMO-WT or the ubiquitin-binding deficient mutant NEMO-F312A-L329P. The bar chart displays the mean percentage and SEM from three independent experiments of Annexin V staining cells after 24 h of culture in vehicle, 10 µM zVAD, 30 µM Necrostatin-1 or a combination of zVAD and Necrostatin-1 either in the presence or absence of 10 ng/ml TNF for 24 h, * denotes p<0.05. The data demonstrates that NEMO-deficient and NEMO-F312A-L329P cells preferentially undergo apoptosis and caspase blockade is required for entry into RIP1 kinase-dependent programmed necrosis. (TIF) [file pone.0041238.s006.tif]

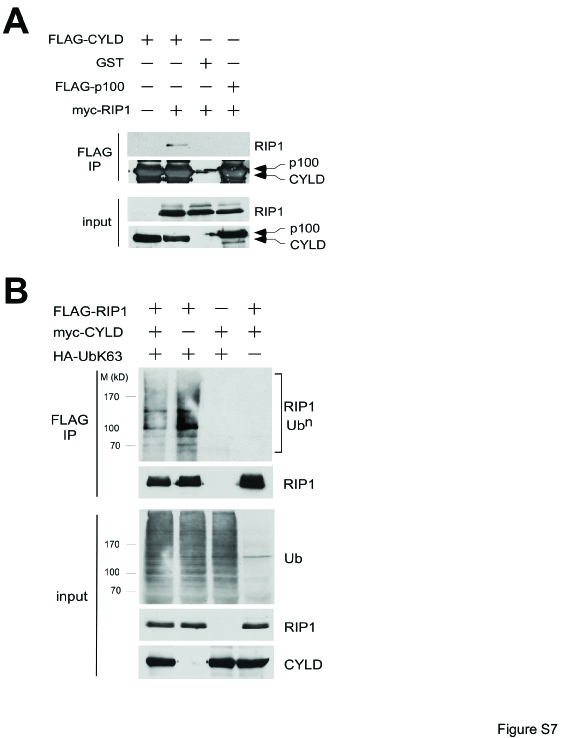

Supplement: Figure S7 — CYLD binds RIP1 and reduces RIP1 ubiquitination. (A) HEK 293 cells were transfected with myc-RIP1 together with FLAG-CYLD, GST or FLAG-p100, with the latter two as negative controls. FLAG-CYLD and FLAG-p100 were immunoprecipitated from triton-soluble lysates and immunoblotted with myc and FLAG-specific antibodies. A sample of lysate was blotted for myc and FLAG to show equivalent expression of each construct. (B) HEK 293 cells were transfected with HA-ubiquitin that contains only lysine 63, FLAG-RIP1 and myc-CYLD. FLAG-RIP1 was immunoprecipitated after SDS-denaturation of triton-soluble lysates and blotted with antibody specific for HA to detect ubiqutinated RIP1. A sample of the lysate was blotted for HA, FLAG and myc to detect transfected ubiquitin, RIP1 and CYLD, respectively. (TIF) [file pone.0041238.s007.tif]
